# Spotlight on USP30: structure, function, disease and target inhibition

**DOI:** 10.3389/fphar.2025.1629709

**Published:** 2025-08-22

**Authors:** Jiapeng Du, Yiyang Gao, Guoqing Xue, Zhuoyue Zhao, Ying Yang, Peng Chu, Xingping Duan

**Affiliations:** ^1^ College of Pharmacy, Dalian Medical University, Dalian, China; ^2^ Neurological Intensive Care Unit, Affiliated Hosp 2, Dalian Medical University, Dalian, China; ^3^ Department of Pharmacy, Zigong Maternal and Child Health Care Hospital, Zigong, China

**Keywords:** USP30, target inhibition, disease, signaling pathway, structure

## Abstract

This review comprehensively summarizes the current understanding of ubiquitin-specific protease 30 (USP30), covering its structural characteristics, functions in cellular processes, associations with diseases, diagnostic and therapeutic strategies, as well as controversies and future perspectives. USP30, a deubiquitinating enzyme, plays crucial roles in mitochondrial quality control, autophagy regulation, and cellular homeostasis. It is implicated in the progression of several malignancies, including hepatocellular carcinoma, breast carcinoma, and glioblastoma, as well as neurodegenerative disorders such as Parkinson’s disease. This involvement is mediated through its regulation of mitochondrial autophagy, stabilization of oncoproteins like Snail and c-Myc, and facilitation of metabolic reprogramming. Inhibition of USP30 has demonstrated potential in reversing the malignant phenotype of tumors and enhancing neuroprotection, highlighting its promise as a versatile therapeutic target. Pharmacological inhibition of USP30, using agents such as S3, MF-094, and FT3967385, enhances ubiquitination and reactivates mitophagy, indicating potential therapeutic benefits in preclinical models. The development of USP30-targeted therapies holds promise but also faces challenges. Further research on USP30 is expected to provide new insights into disease mechanisms and therapeutic interventions.

## 1 Introduction

**FIGURE 1:**
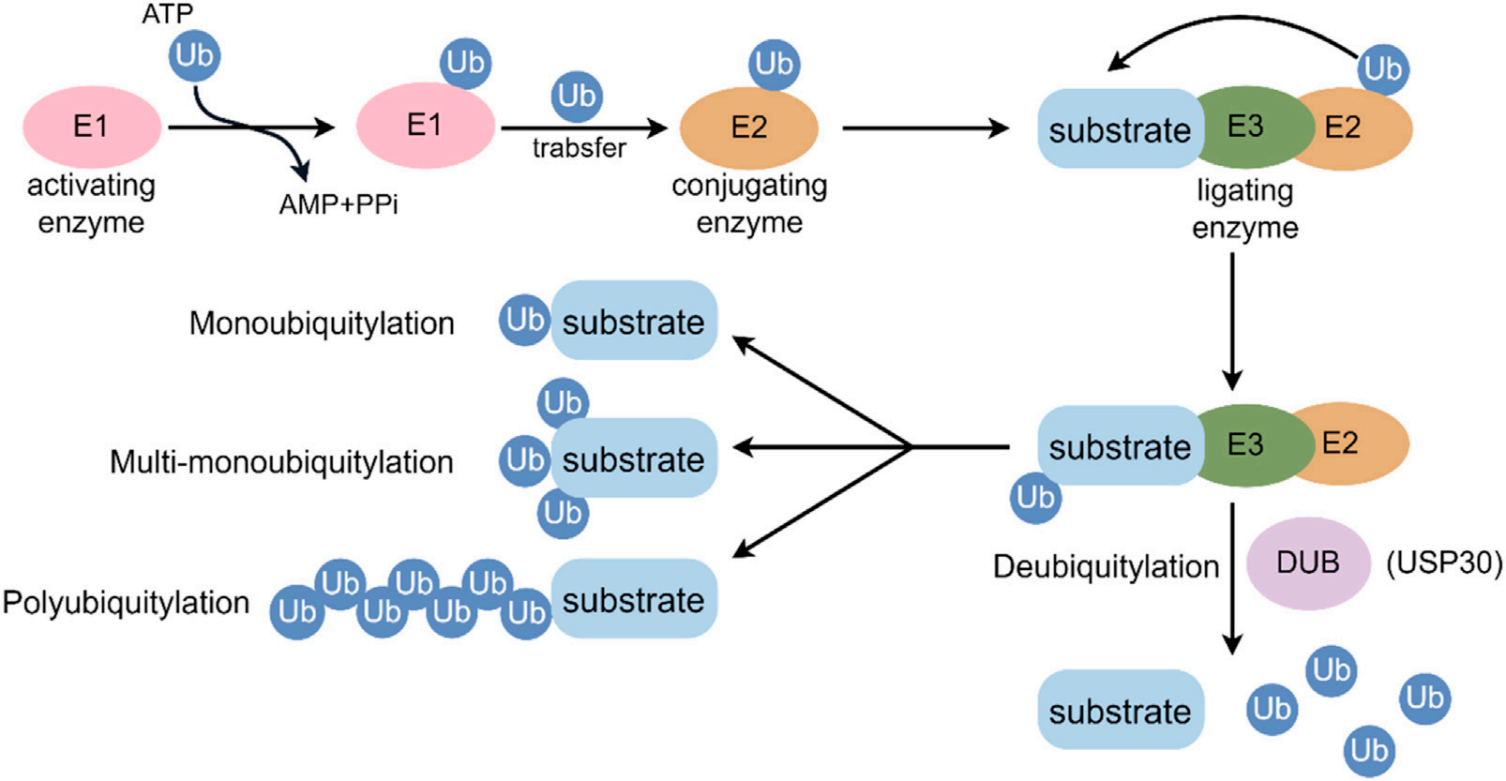
Key events in the ubiquitylation and deubiquitylation process. E1: ubiquitin-activating enzyme; E2: ubiquitin-conjugating enzyme; E3: ubiquitin-protein ligating enzyme; Ub: ubiquitin.

Ubiquitin (Ub) is an evolutionarily conserved protein that marks other proteins for degradation after they have been translated (Popovic et al., 2014). In post-translational modifications, ubiquitin, a 76-amino acid protein, highly conserved protein, is covalently bound to the target substrate in order to regulate the function of the protein (Heap et al., 2017). Most often, ubiquitin is conjugated to lysine residues in target proteins through an enzymatic cascade that involves the E1 activating enzyme, E2 conjugating enzyme, and E3 ligating enzyme (Leznicki and Kulathu, 2017). Initially, Ub is activated by E1 and subsequently transferred to an E2 conjugating enzyme. Afterward, E3 Ub ligases concurrently bind with a Ub-loaded E2 and the substrate protein, mediating the creation of an isopeptide bond between the C terminus of Ub and a substrate lysine (Popovic et al., 2014). Ubiquitin can be conjugated to target proteins either as a single molecule (monoubiquitylation) or as polymeric chains (polyubiquitination) (Melo-Cardenas et al., 2016), each mode having different functional outcomes, including degradation, alteration of function, or cellular localization (Saha et al., 2023) (Figure 1). Ubiquitin is characterized by its seven lysine residues (K6, K11, K27, K29, K33, K48, K63), all of which can be ubiquitinated to form isopeptide chains. A Met1-linked or ‘linear’ chain is formed when ubiquitin is attached to a second ubiquitin’s N-terminus (Swatek et al., 2016). Ubiquitylation is mainly responsible for protein degradation by the 26S proteasome, but it also regulates transcription and DNA repair, cell cycle control, inflammation, and apoptosis (Mennerich et al., 2019).

Deubiquitylating enzymes (DUBs) can hydrolyze the C-terminal bond of Ub by hydrolyzing peptides, amides, esters or thiols, including the post-translationally formed branched peptide bonds in mono- or multi-ubiquitylated conjugates (Singhal et al., 2008). Approximately 100 deubiquitinating enzymes (DUBs) have been identified in humans, classified into six families: ubiquitin-specific proteases (USPs), ubiquitin carboxy-terminal hydrolases (UCHs), ovarian-tumor proteases (OTUs), JAMM/MPN domain-associated metallopeptidases (JAMMs), Machado-Joseph disease protein domain proteases, and monocyte chemotactic protein-induced protein (MCPIP) (Reyes-Turcu et al., 2009). In terms of DUB families, USPs comprise more than half of the known DUBs. These members exhibit high conservation and comprise three subdomains structurally analogous to the fingers, thumb, and palm of a right hand (Melo-Cardenas et al., 2016). Recently, DUB sub-families have gained research attention for their specific deubiquitination preferences and vital roles in biological processes, linking them to diseases like cancer, cardiovascular issues, inflammatory disorders, and neurodegenerative conditions (Popovic et al., 2014; Melo-Cardenas et al., 2016; Singhal et al., 2008; Reyes-Turcu et al., 2009; Huang et al., 2023; Liu et al., 2025; Chen S. et al., 2021).

**FIGURE 2:**
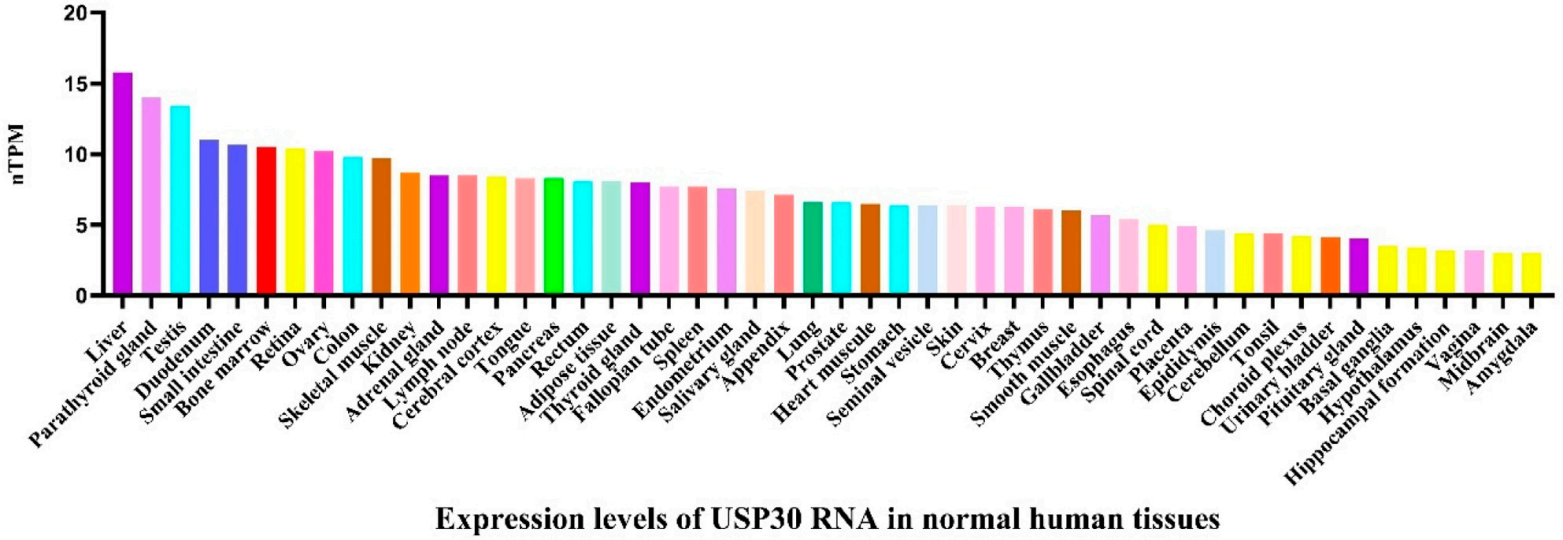
The expression level of USP30 mRNA in normal human tissues. Data derived from the Human Protein Atlas database (https://www.proteinatlas.org/). The image depicts USP30 mRNA expression in a variety of tissues including liver, small intestine, bone marrow, blood vessels, and muscle tissues, indicating a broad distribution across multiple organ systems. The consensus dataset consists of normalized expression (nTPM) levels for 55 tissue types, created by combining the HPA and GTEx transcriptomics datasets using the internal normalization pipeline.

Ubiquitin specific protease 30 (USP30), a member of the USPs, is located at chromosomal locus 12q24.11 and consists of 18 exons. It is highly expressed in human skeletal muscle, cardiac, hepatic, and renal tissues, as well as in tumor tissues such as liver cancer and ovarian cancer (Figure 2). As an outer mitochondrial membrane-anchored protein, USP30 participates in various biological processes by regulating mitochondrial morphology and mitophagy. Studies have shown that USP30 promotes the proliferation of hepatocellular carcinoma cells by positively regulating dynamin-related protein 1 (DRP1)-related mitochondrial fission (Gu et al., 2018) and inhibits apoptosis in leukemia cells by suppressing Parkin-mediated mitophagy and enhancing protein kinase B/mechanistic target of rapamycin (AKT/mTOR) activity (Zhang R. et al., 2022). In the context of Parkinson’s disease treatment, inhibiting USP30 increases translocase of outer mitochondrial membrane 20 (TOM20) ubiquitination and promotes mitophagy, offering a potential therapeutic strategy for Parkinson’s disease caused by PTEN-induced kinase 1 (PINK1) or Parkin mutations (Fang et al., 2023). Therefore, targeted inhibition of USP30 plays important roles in promoting autophagy, suppressing mitochondrial fission, protecting dopaminergic neurons, and inhibiting cancer cell survival and proliferation.

In this review, we comprehensively outline the structural and molecular features of USP30, along with its involvement in a range of biological processes, including mitophagy and apoptosis. Additionally, we explore the role of USP30 in various diseases, such as neurodegenerative disorders, peroxisome biogenesis disorders, and cancer, as well as the regulatory functions of USP30-AS1 in cancer progression and its potential as a prognostic biomarker for different cancers. Finally, we summarize the current landscape of targeted inhibitors against USP30, aiming to provide new insights and strategies for the treatment of related diseases.

## 2 Structural and functional characterization of USP30

### 2.1 Structural characteristics of USP30

**FIGURE 3:**
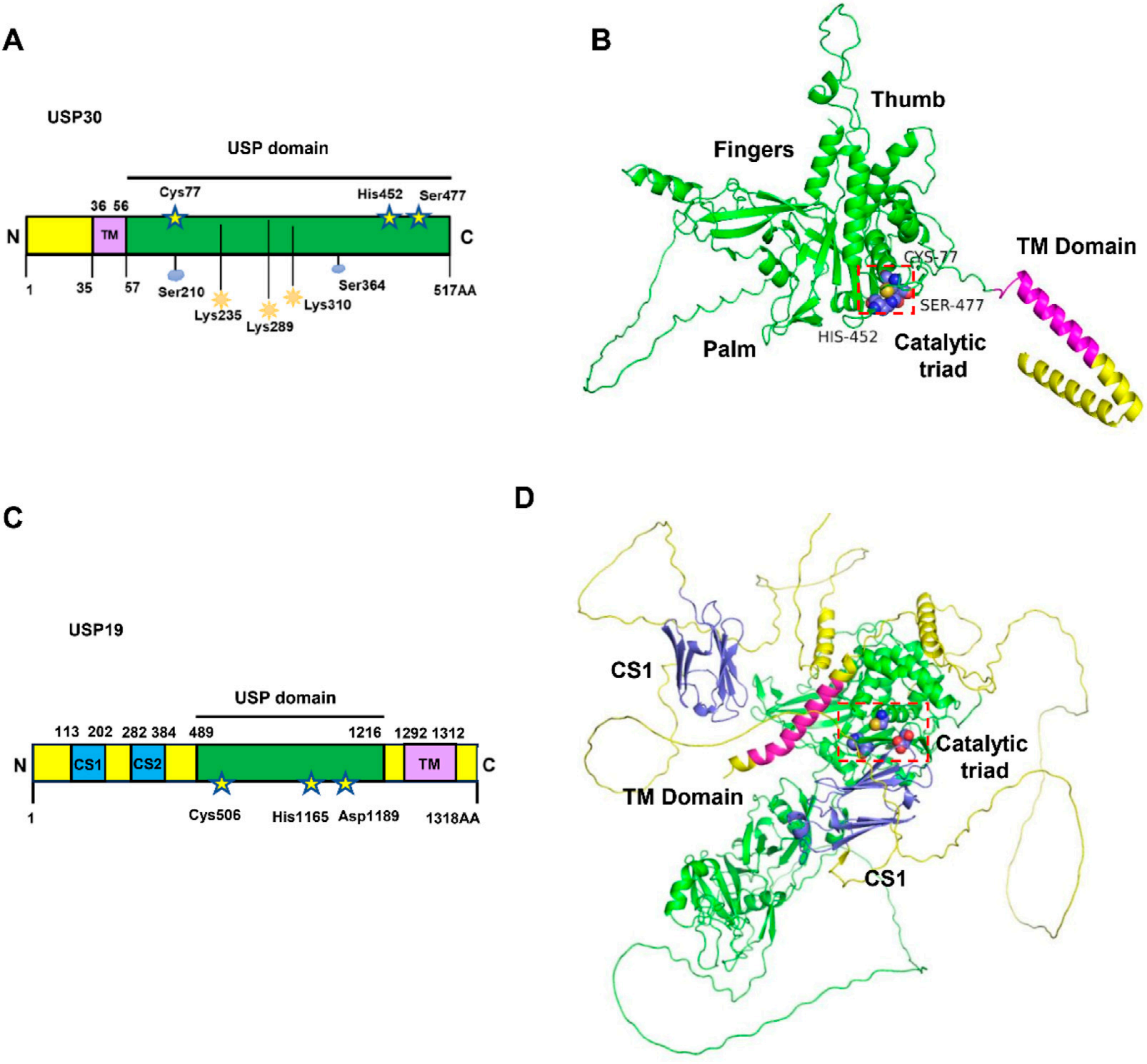
The structure and domains of USP30. **(A)** Schematic representation of protein domain architecture of USP30. The positions of the amino acids Cys77, His452 and Ser477 in the catalytic triad are indicated and the sequences of transmembrane (TM) domain of USP30 are listed. **(B)** The AlphaFold3-predicted structure of the full-length USP30 is depicted, with the catalytic triad illustrated as blue spheres utilizing PyMOL. The USP domain is represented by a green cartoon, while the TM domain is depicted as a magenta cartoon. **(C)** Schematic representation of protein domain architecture of USP19. The positions of the amino acids Cys506, His1165 and Asp1189 in the catalytic triad are indicated and the sequences of TM domain of USP19 are listed. **(D)** AlphaFold3-predicted structure of full length USP19. The catalytic triad is shown as blue spheres using PyMOL.

As a distinctive member of the deubiquitinase family, human USP30 exhibits unique structural features that set it apart from typical cytoplasmic or nuclear counterparts. While most deubiquitinases exist as soluble proteins, USP30 and its paralog USP19, represent rare exceptions with transmembrane domains. USP19 contains two CS (CHORD-SGT1)/P23 domains at its N-terminus, which potentially interact with the Heat Shock Protein 90 (HSP90) chaperone, as well as a central USP domain responsible for its deubiquitinating activity. In contrast, USP30 exhibits a more complex distribution pattern (Figures 3A–D) (Bingol et al., 2014). Immunohistochemical studies consistently demonstrate its predominant localization on outer mitochondrial membranes and peroxisomes, with detectable presence in cytoplasmic and nuclear compartments under specific conditions (Hassink et al., 2009). This multifaceted localization reflects its tripartite domain organization: an N-terminal mitochondrial targeting sequence (residues 1–35), a central transmembrane anchor (residues 36–56), and a C-terminal catalytic USP domain (residues 57–517). The transmembrane domain serves as a molecular clamp, securing USP30 to organelle membranes while allowing functional flexibility through its exposed catalytic domain (Figures 3A,B) (Wauer et al., 2015a).

Crystallographic breakthroughs have illuminated USP30’s molecular architecture through multiple resolved structures. The 5OHK (2.34Å) and 5OHN (3.60Å) structures capture USP30 in complex with ubiquitin propargylamide, revealing conserved interactions with ubiquitin’s hydrophobic patch. Notably, the 5OHP structure (2.80 Å resolution) demonstrates USP30’s specific engagement with K6-linked diubiquitin, while 8D0A and 8D1T structures (3.19 Å and 2.94 Å respectively) showcase inhibitor-binding modes (Wauer et al., 2015b). Complementing experimental data, AlphaFold predictions corroborate the spatial arrangement of its catalytic core. At the center of USP30’s enzymatic activity lies an unconventional catalytic triad—Cys77, His452, and Ser477—a configuration distinct from the classical Cys-His-Asp/Asn triad observed in most USP family members. This unique triad collaborates with adjacent structural elements to confer precise recognition of K6-linked ubiquitin chains (Gersch et al., 2017; Sato et al., 2017).

The molecular choreography of USP30’s ubiquitin recognition involves two cooperative binding sites. The primary S1 site, formed by finger, thumb, and palm subdomains, establishes robust interactions with distal ubiquitin through β-strand-mediated hydrogen bonds. A secondary S1 site, though exhibiting weaker binding affinity, selectively engages proximal ubiquitin in K6-linked chains *via* hydrophobic contacts centered on the Phe4 residue (Gersch et al., 2017; Sato et al., 2017). Structural analyses identify conserved residues His445, His452, and Trp475 as critical mediators of these interactions, creating a geometrically constrained catalytic pocket that preferentially accommodates K6-linked ubiquitin topology. This spatial discrimination mechanism underlies USP30’s remarkable specificity, with biochemical assays demonstrating over fivefold higher cleavage efficiency for K6-linked *versus* K48-linked chains (Bingol et al., 2014; Gersch et al., 2017; Ye et al., 2009).

USP30’s enzymatic activity operates within a dynamic regulatory network. Mitochondrial stress sensors PINK1 and Parkin emerge as key modulators, with their expression levels directly influencing USP30’s deubiquitination efficiency (Bingol et al., 2014; Liang et al., 2015). Pharmacological studies further reveal susceptibility to covalent inhibitors, as evidenced by structural snapshots of inhibitor-bound states (8D0A, 8D1T) (Wauer et al., 2015b). When contextualized within the broader DUB family, USP30’s functional niche becomes apparent: while OTU domain-containing ubiquitin aldehyde-binding protein 1 (OTUB1) specializes in K48-linked chain processing for proteasomal degradation and OTUB2 modulates K63-linked chains in inflammatory signaling, USP30 occupies a unique position in mitochondrial quality control through its K6 chain preference (Komander and Rape, 2012; Clague et al., 2019).

In essence, USP30 embodies three defining characteristics: its membrane-anchored operational platform enables organelle-specific regulation; the evolutionarily divergent catalytic triad provides mechanistic specialization for K6 chain processing; and context-dependent modulation integrates its activity into cellular stress response pathways. These structural-functional insights establish USP30 as a molecular rheostat fine-tuning mitochondrial ubiquitination landscapes, a concept we will further explore in subsequent discussions of its pathophysiological roles.

### 2.2 Evolution of USP30 structural studies

The investigation of USP30’s structure has progressed significantly over time, driven by the goal of elucidating its molecular function. Initial studies concentrated on pinpointing its cellular localization and characterizing its fundamental enzymatic activities (Nakamura and Hirose, 2008). With advancements in technology, X-ray crystallography emerged as a powerful tool, allowing researchers to determine the detailed crystal structure of USP30 in complex with ubiquitin chains. This provided valuable insights into how USP30 recognizes its substrates and executes the cleavage process (Sato et al., 2017).

In more recent years, the combination of structural biology, quantitative proteomics, and biochemical assays has become instrumental in understanding how USP30 inhibitors work (O'Brien et al., 2025). For instance, by examining how covalent and non-covalent inhibitors bind to USP30, researchers have uncovered distinct interaction patterns. Covalent inhibitors, such as USP30-I-1, which contain a cyanopyrrolidine reactive group, form strong bonds with regions around the catalytic cysteine and create a binding pocket within the thumb and palm domains of the protein (O'Brien et al., 2025). In contrast, non-covalent inhibitors bind in a slightly different manner, closer to the active site cysteine (Cys77). These discoveries have laid the groundwork for enhancing the selectivity of inhibitors, specifically targeting USP30 while minimizing effects on other members of the deubiquitinating enzyme (DUB) family.

### 2.3 Techniques for analyzing USP30 structure

A variety of techniques have been employed to analyze the structure of USP30. X-ray crystallography has been a key technique, enabling the determination of the three-dimensional structure of USP30 in complex with ubiquitin chains at high resolution (Sato et al., 2017). This technique has provided detailed information about the active site, substrate-binding regions, and the overall architecture of the protein.

In addition, AlphaFold is also an important tool for predicting the structure of USP30. AlphaFold is a series of artificial intelligence programs developed by DeepMind. It analyzes the amino acid sequences of proteins and predicts how these sequences interact to form the three-dimensional structure of the proteins. By leveraging a vast amount of sequence information and co-evolutionary data, AlphaFold enhances the accuracy of structural predictions. This approach not only improves prediction accuracy but also provides new research tools for the fields of biology and medicine. We can access the AlphaFold Database or use platforms such as AlphaFold Server and AlphaFold2 TIB Server to perform AlphaFold predictions. It is important to note that AlphaFold’s predictions are not infallible. It may face challenges when dealing with certain types of proteins or when there is insufficient homologous sequence information. Moreover, the protein structures predicted by AlphaFold are typically static, while actual proteins are dynamically changing within living organisms (Abramson et al., 2024; Jumper et al., 2021; Yang et al., 2023).

## 3 USP30 in cellular function

### 3.1 USP30 in mitochondrion

Mitochondria, as central organelles for cellular energy metabolism and signaling regulation, play a decisive role in maintaining cellular homeostasis through their quantity and functional integrity (Acin-Perez et al., 2011). To ensure efficient operation of mitochondrial quality control systems, cells have evolved two synergistic mechanisms: mitochondrial dynamics (Chan, 2020) and mitophagy (Onishi et al., 2021). Mitochondrial dynamics regulate organelle morphology and distribution through a dynamic balance of continuous fission and fusion: DRP1 facilitates mitochondrial fission by constricting the mitochondrial membrane (Jin et al., 2021), while mitofusin 1/2 (MFN1/2) mediates outer membrane fusion by tethering adjacent mitochondria (Sack, 2011). Complementing this process, mitophagy selectively identifies and degrades damaged mitochondria *via* the PINK1-Parkin ubiquitination cascade. Upon mitochondrial depolarization, the kinase PINK1 accumulates on the outer mitochondrial membrane (OMM) to activate Parkin, an E3 ubiquitin ligase, which triggers autophagosomal engulfment through ubiquitin-dependent signaling (Table 1) (Li et al., 2023).

**TABLE 1:** Substrates of USP30 in cellular and biological processes.

Substrate	Mechanism summary	Related cellular biology process	References
OMM (Miro1, TOM20, and 41 other Parkin ubiquitination substrates negatively regulated by USP30)	Negatively regulate PINK1-Parkin-dependent mitophagy	Mitophagy	Bingol et al. (2014)
Ubiquitin Chains	Cleave ubiquitin chains constructed by Parkin at Lys6, Lys11, and Lys48 sites		Cunningham et al. (2015)
DRP1	Promote DRP1-associated mitochondrial fission	Mitochondrial dynamics	Gu et al. (2018)
MFN1/2	Attenuate the ubiquitination levels of the nondegradation pathways of MFNs		Chen et al. (2022)
MFN2	Inhibit the ubiquitination and degradation of MFN 2		Chen et al. (2021b)
PEX2	Inhibit pexophagy by neutralizing the E3 ubiquitin ligase function of PEX2	Pexophagy	Marcassa et al. (2018), Yue et al. (2014)
PEX5 and PMP70	Deubiquitinate PEX5 and PMP70		Marcassa et al. (2018), Yue et al. (2014)
Parkin	Antagonize the activity of Parkin to sustain AKT/mTOR activity and inhibit apoptosis	Apoptosis and cell death	Zhang et al. (2022a)
BAX/BAK	Control BAX/BAK-dependent apoptosis		Yan et al. (2021)
NLRP1	Disrupt mitophagy and activates NLRP1 inflammasome-mediated neuroinflammation	Inflammation	Gao and Huang (2024)
NLRP3	Activate the NLRP3 inflammasome by deubiquitinating NLRP3		Kluge et al. (2018), Li et al. (2022b)
MAVS	CDK5-USP30-MAVS Pathway		Jenab et al. (2020)
GNPAT	Interact with GNPAT to prevent the degradation of DRP1	Cancer	Gu et al. (2018)
TOMM40	Deubiquitinating TOMM40 promotes the development of breast cancer		Gao X. et al. (2025)
Snail	Promote the EMT program of breast cancer cells by stabilizing Snail		Sun et al. (2024)
c-Myc	Increase c-Myc protein levels, leading to OSCC development		Gomez et al. (2023)

Recent studies highlight the critical regulatory roles of deubiquitinating enzymes (USPs) in this quality control network. For instance, USP8 and USP13 promote mitophagy by directly removing ubiquitin modifications from Parkin, whereas USP15, USP30, USP33, and USP35 counteract Parkin-mediated ubiquitination of OMM proteins to suppress mitophagy (Luo et al., 2021). Notably, USP30, localized to the OMM, exhibits dual regulatory functions in both mitochondrial dynamics and mitophagy. On one hand, it stabilizes fusion proteins (e.g., MFN1/2) through deubiquitination (Sack, 2011), thereby maintaining mitochondrial fusion capacity. On the other hand, it attenuates mitophagy by cleaving Parkin-deposited ubiquitin chains, effectively blocking autophagic signal propagation (Liang et al., 2015). Such dual functionality positions USP30 as a key molecular node bridging mitochondrial morphological plasticity and quality surveillance.

#### 3.1.1 USP30 in mitophagy

The deubiquitinating enzyme (DUB) activity of USP30 critically regulates mitochondrial homeostasis by counteracting Parkin-mediated ubiquitination of mitochondrial proteins, thereby suppressing mitophagy and preserving mitochondrial integrity. This regulatory function positions USP30 as a potential therapeutic target for diseases linked to mitochondrial dysfunction. Central to this process are PINK1 and Parkin, two Parkinson’s disease-associated proteins that orchestrate the polyubiquitination of damaged mitochondrial surface proteins to initiate autophagic clearance (Cunningham et al., 2015).

Under physiological conditions, mitochondrial depolarization triggers PINK1 stabilization on the OMM, where it undergoes autophosphorylation and subsequently phosphorylates ubiquitin and Parkin. These post-translational modifications enhance Parkin’s affinity for the OMM, facilitating its cytosolic translocation and initiating a ubiquitination cascade that marks damaged mitochondria for autophagosomal degradation (Li et al., 2023). USP30 antagonizes this process through three distinct yet interconnected mechanisms: (1) It removes ubiquitin tags from OMM proteins, dampening Parkin recruitment and activation (Luo et al., 2021); (2) It directly inhibits Parkin-mediated ubiquitination of key substrates, including TOM20, VDAC isoforms, and Miro1, as evidenced by studies showing that USP30 knockdown amplifies—while its overexpression suppresses—substrate ubiquitination and subsequent autophagic flux (Liang et al., 2015); (3) It selectively hydrolyzes Parkin-synthesized ubiquitin chains, with biochemical assays demonstrating preferential cleavage of K6-, K11-, and K48-linked polyubiquitin structures (Cunningham et al., 2015).

Proteomic analyses have identified 41 Parkin substrates negatively regulated by USP30, underscoring its broad impact on mitochondrial protein homeostasis. Intriguingly, this antagonistic relationship is bidirectional: Parkin itself can promote USP30 degradation *via* the ubiquitin-proteasome system, suggesting a dynamic equilibrium between ubiquitination and deubiquitination in mitophagy regulation. This reciprocal interaction implies a finely tuned feedback loop that may adapt to cellular stress levels, though the molecular details of this crosstalk remain to be fully elucidated (Bingol et al., 2014).

Emerging therapeutic strategies exploit USP30’s regulatory role. For instance, Chen and colleagues developed a nanoparticle system co-delivering USP30-targeting small interfering RNA (siRNA) and PINK1 antibodies to selectively enhance clearance of irreversibly damaged mitochondria. This approach demonstrated efficacy in both cellular and animal models, highlighting the translational potential of modulating the USP30-PINK1/Parkin axis to mitigate mitochondrial damage in pathological contexts (Chen et al., 2022).

By balancing ubiquitination dynamics and mitochondrial protein stability, USP30 serves as a critical rheostat in mitochondrial quality control, bridging organelle homeostasis with cellular adaptability. Future studies dissecting its context-dependent interactions and hierarchical regulation will deepen our understanding of mitophagy and inform precision therapies for mitochondrial disorders.

#### 3.1.2 USP30 in regulating the mitochondrial dynamics

Mitochondrial dynamics, governed by a balance between fission and fusion processes, are orchestrated by key regulatory proteins. DRP1, a transmembrane GTP hydrolase (GTPase) localized to the outer mitochondrial membrane, serves as a central driver of mitochondrial and peroxisomal membrane fission. Structurally, DRP1 contains an N-terminal GTPase domain, a bundle signaling element, a middle domain, and a GTPase effector domain. Beyond its physiological roles, dysregulated DRP1 activity has been implicated in neurodegenerative diseases, aging, and cancer, with emerging evidence highlighting its contribution to tumor metastasis (Rochon et al., 2024). Notably, USP30, a deubiquitinating enzyme, enhances DRP1-mediated mitochondrial fission by promoting its K48-linked deubiquitination in a Glyceronephosphate O-acyltransferase (GNPAT)-dependent manner. In hepatocellular carcinoma (HCC) models (Hep3B and HepG2 cells), inhibition of either GNPAT or USP30 significantly reduces DRP1 protein levels, triggering mitochondrial fusion and suppressing cancer cell proliferation and HCC progression. These findings position USP30 as a potential therapeutic target for HCC (Gu et al., 2018).

In contrast, mitofusins MFN1 and MFN2, transmembrane GTPases that form homo- or heterodimers on the outer mitochondrial membrane, are essential for mitochondrial fusion (Li et al., 2019). The regulatory role of USP30 in mitochondrial fusion exhibits striking cell type specificity. Nakamura et al. demonstrated that USP30 depletion in HeLa cells induces elongated mitochondrial networks *via* enhanced MFN1/MFN2 activity, without altering the expression levels of mitochondrial dynamics regulators. This phenotype was reversed by co-knockdown of MFN1/2, which caused mitochondrial fragmentation, suggesting USP30 normally suppresses mitofusin activity to restrain fusion (Nakamura and Hirose, 2008). Paradoxically, USP30 also stabilizes MFN2 under stress conditions. In SK-N-BE (2) neuroblastoma cells subjected to oxygen-glucose deprivation/reperfusion (OGDR) injury, MFN2 undergoes rapid ubiquitination and degradation, leading to mitochondrial fragmentation. USP30 overexpression in this context inhibits MFN2 ubiquitination, preserves its protein levels, and prevents fission, implicating USP30 as a protective modulator of mitochondrial fusion during ischemic stress (Chen C. et al., 2021).

The dual regulatory mechanisms of USP30 are further illustrated by its interaction with small-molecule inhibitors. Compound S3, a covalent inhibitor targeting a catalytic cysteine residue in USP30, suppresses its deubiquitinase activity. In retinal ganglion cells (RGCs), S3 treatment or USP30 knockdown increases non-degradative ubiquitination of MFN1/2, enhancing their fusogenic activity. This finding aligns with the model that USP30 constitutively dampens mitofusin function through deubiquitination (Zhuang et al., 2022).

Collectively, these studies reveal a context-dependent duality in USP30’s regulation of mitochondrial dynamics: (1) It promotes DRP1-driven fission in HCC by counteracting K48-linked ubiquitination, and (2) modulates MFN1/2-mediated fusion through both inhibitory and stabilizing mechanisms depending on cellular stress conditions. This functional ambivalence—suppressing mitofusin activity under basal conditions while protecting MFN2 from stress-induced degradation—underscores the cell type- and stimulus-specific nature of USP30’s roles. Such complexity necessitates precise therapeutic targeting strategies, whether for inhibiting USP30 to curb HCC progression or enhancing its activity to mitigate cerebral ischemia-reperfusion injury. Further investigation is required to delineate the molecular switches governing USP30’s opposing functions across physiological and pathological contexts.

### 3.2 USP30 in peroxisome

Peroxisomes are ubiquitous eukaryotic organelles essential for metabolic processes, including the β-oxidation of very long-chain and branched fatty acids, hydrogen peroxide detoxification, and the biosynthesis of specialized lipids such as bile acids, ether phospholipids, and isoprenoids (Wanders and Waterham, 2006; Smith and Aitchison, 2013). These organelles share functional and regulatory parallels with mitochondria, including roles in lipid metabolism, reactive oxygen species (ROS) management, and reliance on selective autophagy (mitophagy and pexophagy, respectively) for quality control (Islinger et al., 2018). Unlike its role in mitochondrial autophagy, USP30 exhibits a distinct regulatory function in peroxisomal homeostasis, operating independently of the PINK1-Parkin pathway to modulate basal pexophagy (Bingol et al., 2014; Marcassa et al., 2018).

Pexophagy (the selective degradation of peroxisomes) is tightly regulated under stress conditions such as nutrient deprivation or hypoxia (Cho et al., 2018). During amino acid starvation, mTOR inhibition stabilizes the E3 ubiquitin ligase peroxisomal biogenesis factor 2 (PEX2) on peroxisomal membranes. PEX2 subsequently ubiquitinates peroxisomal membrane proteins such as PEX5 and 70 kDa Peroxisomal Membrane Protein (PMP70), marking peroxisomes for autophagic clearance *via* recruitment of autophagy receptors NBR1 and p62 (Deosaran et al., 2013; Sargent et al., 2016). USP30 counteracts this process by antagonizing PEX2-mediated ubiquitination. Overexpression of USP30 preserves peroxisome numbers under both basal and stress conditions, likely through deubiquitination of PEX5 and PMP70, thereby blunting pexophagy. This regulatory axis positions USP30 as a critical suppressor of excessive peroxisomal turnover (Table 1) (Marcassa et al., 2018; Waterham and Ebberink, 2012).

Dysregulated pexophagy contributes to diverse pathologies. In peroxisome biogenesis disorders (PBDs)—caused by mutations in PEX genes (e.g., PEX1, PEX6, PEX26)—defective peroxisomal AAA ATPase activity leads to aberrant accumulation of ubiquitinated PEX5 and unchecked pexophagy, exacerbating peroxisome loss (Waterham and Ebberink, 2012; Fujiki et al., 2014). USP30 mitigates this pathology by deubiquitinating PEX5, restoring peroxisomal stability, and highlighting its potential as a therapeutic target for PBDs (Marcassa et al., 2018). Beyond PBDs, aberrant pexophagy is implicated in neurodegenerative diseases (e.g., Alzheimer’s disease, amyotrophic lateral sclerosis), metabolic syndrome, cirrhosis, and cancers (e.g., hepatocellular carcinoma, breast cancer) (Wanders and Waterham, 2006; Fransen et al., 2017; Pratama et al., 2024). However, whether USP30 modulation could ameliorate these conditions remains speculative, necessitating further investigation into its cell type- and context-dependent roles.

While USP30 emerges as a key regulator of peroxisomal integrity, critical gaps persist. Its precise molecular interplay with PEX2 and other pexophagy mediators requires elucidation. Furthermore, the therapeutic window for USP30 modulation—balancing its anti-pexophagy effects against potential risks of peroxisomal overaccumulation—must be rigorously assessed. Addressing these questions will clarify USP30’s viability as a target for peroxisome-associated diseases.

### 3.3 USP30 in apoptosis and cell death

Apoptosis, a programmed cellular self-destruction mechanism, plays essential roles in developmental morphogenesis, tissue homeostasis, and elimination of damaged cells (Elmore, 2007). Mitochondria serve as central regulators of cell death, particularly through the intrinsic apoptosis pathway characterized by mitochondrial regulation and non-receptor-mediated initiation (Bock and Tait, 2020). Emerging evidence identifies USP30 as a key modulator of apoptotic processes, with concurrent development of selective small-molecule activity-based probes for monitoring USP30 function (Table 1).

The AKT/mTOR signaling pathway, a critical intracellular regulatory network governing cell growth, proliferation, metabolism, and survival, demonstrates functional interactions with USP30 (Sun et al., 2020). Mitochondrial stress activates this pathway, which can be modulated by the Parkin-USP30 axis. Parkin suppresses AKT/mTOR signaling during mitophagy, correlating with downregulation of downstream effectors Eukaryotic translation initiation factor 4E-binding protein 1 (4EBP1) and Ribosomal protein S6 kinase (P70S6K). USP30 overexpression counteracts Parkin-mediated suppression, maintaining AKT/mTOR activity and inhibiting apoptosis. Pharmacological USP30 inhibition reduces AKT levels in both HeLa Parkin USP30 cells and Jurkat T leukemia cells under mitochondrial stress or chemotherapy, promoting apoptosis. Chemical screening data suggest potential therapeutic synergy between USP30 inhibitors and AKT/mTOR pathway inhibitors for leukemia treatment, positioning USP30 as a novel therapeutic target requiring further investigation in combinatorial regimens (Zhang R. et al., 2022).

USP30 exerts regulatory control over BCL2 associated X/BCL2 antagonist/killer 1 (BAX/BAK)-dependent apoptosis, the central execution mechanism for MOMP (mitochondrial outer membrane permeabilization). In human osteosarcoma (U2-OS) and breast cancer (MCF7) models, USP30 depletion enhances cancer cell sensitivity to pro-apoptotic BH3 mimetics, revealing its anti-apoptotic role (Liang et al., 2015). The USP30 inhibitor auranofin demonstrates therapeutic potential by increasing BAX ubiquitination and mitochondrial localization, inducing BAX-dependent apoptosis in lung cancer cells. This establishes USP30 inhibition as a promising strategy for combinatorial cancer therapies beyond its established role in Parkinson’s disease (Yan et al., 2021).

Experimental evidence reveals context-dependent effects of USP30 modulation: USP30 overexpression inhibits apoptosis, while its genetic or pharmacological inhibition paradoxically reduces cell death in specific pathological contexts. USP30 ablation mitigates oxidative stress and neural apoptosis post-traumatic brain injury (Wu et al., 2023). In ototoxic models, USP30 knockdown prevents neomycin-induced hair cell apoptosis, evidenced by increased viable hair cells and reduced TUNEL-positive cells (Zhang et al., 2023). Neuronal culture studies demonstrate that USP30 inhibition promotes mitochondrial quality control through damaged organelle segregation and mitophagy induction (Liu et al., 2023). In Alzheimer’s disease models, miR-137-5p-mediated USP30 suppression reduces hippocampal and cortical neuronal apoptosis, effects partially reversible by USP30 overexpression (Jiang et al., 2023).

### 3.4 USP30 in inflammation

Inflammation is a protective biological response to harmful stimuli, such as pathogens, cellular damage, or irritants, functioning to eliminate threats and initiate tissue repair (Jenab et al., 2020). Emerging evidence suggests USP30 participates in inflammatory regulation through distinct molecular mechanisms.

Sevoflurane (Sevo), a volatile anesthetic, exacerbates neurotoxicity by upregulating USP30, which disrupts mitophagy and activates NOD-like-receptor containing a pyrin domain 1 (NLRP1) inflammasome-mediated neuroinflammation (Gao and Huang, 2024). This highlights USP30’s role in linking mitochondrial quality control to inflammatory signaling.

Ren et al. identified the CDK5-USP30-MAVS axis as a critical pathway in Parkinson’s disease (PD) models. In 1-methyl-4-phenyl-1,2,3,6-tetrahydropyridine/1-methyl-4-phenylpyridinium (MPTP/MPP^+^)-induced neurodegeneration, cyclin-dependent kinase 5 (CDK5) phosphorylates USP30 to inhibit mitophagy, thereby activating the mitochondrial antiviral signaling protein (MAVS)-dependent inflammatory cascade. Pharmacological inhibition of CDK5 suppressed USP30 upregulation, attenuated MAVS pathway activation, and exerted protective effects in both *in vitro* (MPP+ -treated neuronal cells) and *in vivo* (MPTP-induced mouse) PD models. These interventions reduced neurodegeneration and alleviated motor deficits, establishing the CDK5-USP30-MAVS pathway as a potential therapeutic target for neuroinflammatory disorders associated with mitochondrial dysfunction (Table 1) (Ren et al., 2024).

## 4 USP30 involvement in disease

Research indicates that USP30 plays a significant role in the pathogenesis of various diseases, including cancers, neurodegenerative disorders, and peroxisome biogenesis disorders (PBDs). Notably, abnormal USP30 expression is frequently observed in multiple malignant tumors and correlates with tumor progression and prognosis.

### 4.1 Cancer

Tumorigenesis involves the inactivation of tumor suppressor genes, activation of oncogenes, dysregulated apoptosis, and aberrant signaling pathways (Weinberg, 1994). DUBs critically regulate many tumor-related mechanisms, and USP30, as a member of the DUB family, has emerged as a key player in cancer development (Bonacci and Emanuele, 2020). USP30 is highly expressed in diverse malignancies, such as hepatocellular carcinoma (HCC), breast cancer (BC), glioblastoma (GBM), colon cancer, acute myeloid leukemia (AML), cervical cancer (CC), oral squamous cell carcinoma (OSCC), bladder urothelial carcinoma (BLCA), ovarian carcinoma (OC), and melanomas. Elevated USP30 levels are closely associated with tumor aggressiveness and serve as an adverse prognostic marker across multiple cancer types (Gu et al., 2021).

#### 4.1.1 Hepatocellular carcinoma (HCC)

Hepatocellular carcinoma (HCC), a leading cause of cancer-related mortality, arises from chronic liver conditions such as obesity, cirrhosis, and hepatitis (Gu et al., 2018). USP30 expression is significantly upregulated in HCC tissues compared to adjacent non-tumor tissues and correlates with Tumor-Node-Metastasis (TNM) staging and clinical prognosis. High USP30 levels enhance the proliferation, invasion, and migration capacities of HCC cells, positioning USP30 as a potential diagnostic and therapeutic target for liver cancer (Gu et al., 2018).

HCC pathogenesis is closely linked to inflammation and dysregulated lipid metabolism (Currie et al., 2013). Genetic amplification or Myc/KDM1A-mediated regulatory networks stimulate GNPAT expression, which interacts with USP30 to stabilize DRP1 by preventing its degradation. This interaction modulates mitochondrial dynamics, lipid metabolism, and hepatocarcinogenesis, highlighting potential therapeutic targets for further exploration (Gu et al., 2018).

Recent studies reveal a novel regulatory axis in HCC involving IκB kinase β (IKKβ)-mediated phosphorylation of USP30 and ATP citrate lyase (ACLY). This phosphorylation promotes ACLY deubiquitination and activation, driving lipid synthesis, inflammation, and HCC progression. USP30 deficiency markedly suppresses these oncogenic processes. Intriguingly, combined inhibition of ACLY and PD-L1, both upregulated by IKKβ, synergistically inhibits HCC development. The IKKβ-USP30-ACLY axis thus represents a critical pathway regulating lipid metabolism, inflammation, and hepatocarcinogenesis. Targeting this axis, particularly through dual ACLY and programmed death ligand 1 (PD-L1) inhibition, presents a promising therapeutic strategy warranting further investigation (Gu et al., 2021).

#### 4.1.2 Breast cancer (BC)

Breast cancer (BC), the most prevalent malignancy in women and the second leading cause of cancer-related deaths globally, is characterized by aggressive subtypes such as triple-negative breast cancer (TNBC) (Chen C. et al., 2021). USP30 expression is significantly dysregulated in BC, particularly in TNBC, and correlates with poor patient prognosis. Functionally, USP30 inhibits the proliferation, migration, and invasion of breast cancer cells. Translocase of outer mitochondrial membrane 40 (TOMM40) has been identified as an important prognostic biomarker for BC. A series of experimental validations have shown that USP30 can interact with TOMM40, and USP30 deubiquitinating TOMM40 promotes the development of breast cancer (Gao X. et al., 2025).

*In vitro* experiments demonstrate that USP30 knockdown suppresses breast cancer cell proliferation and epithelial-mesenchymal transition (EMT), while USP30 overexpression exacerbates these processes. *In vivo* studies further confirm that USP30 depletion inhibits tumor growth. USP30 promotes EMT and chemoresistance in breast cancer by stabilizing Snail protein through deubiquitination, which reduces sensitivity to paclitaxel. These findings underscore USP30 as a potential therapeutic target for BC (Sun et al., 2024).

Notably, a risk assessment model incorporating lncRNA-USP30-AS1 has been developed to predict immunotherapy responses in TNBC patients, independent of expression levels. lncRNA USP30-AS1 exhibits a positive correlation with PD-L1 expression, suggesting its potential as a therapeutic target for TNBC immunotherapy (Li et al., 2024). Additionally, metabolism-associated lncRNAs, including USP30-AS1, may serve as reliable prognostic and therapeutic response biomarkers in BC (Ge et al., 2024).

#### 4.1.3 Glioblastoma (GBM)

Glioblastoma (GBM), the most aggressive astrocyte-derived central nervous system malignancy, is associated with high morbidity, mortality, and recurrence rates. lncRNA USP30-AS1 has emerged as a prognostic biomarker, with elevated expression observed in high-grade gliomas compared to lower-grade counterparts. High USP30-AS1 levels correlate with poor survival in patients with primary or recurrent gliomas, including high-grade tumors.

Functionally, USP30-AS1 disrupts mitochondrial homeostasis by suppressing mitophagy, a process critical for maintaining mitochondrial integrity. Impaired mitophagy contributes to mitochondrial dysfunction, a hallmark of cancer progression. Although USP30 itself regulates mitochondrial quality control, USP30-AS1-mediated suppression of mitophagy occurs independently of USP30 expression, as USP30-AS1 silencing does not alter USP30 levels. This suggests distinct roles for USP30-AS1 in mitochondrial dysregulation and glioma progression, highlighting its potential as a therapeutic target in GBM (Wang et al., 2021).

#### 4.1.4 Colon cancer

Colon cancer remains a leading cause of cancer-related mortality in China and globally, ranking among the most prevalent malignancies worldwide. USP30-AS1 has emerged as a critical biomarker in colon cancer progression and prognosis, demonstrating significant correlations with aggressive clinicopathological features including larger tumor size, lymph node metastasis, and advanced TNM stage, all indicative of poor clinical outcomes (Yang et al., 2019). Notably, USP30-AS1 serves as an independent prognostic indicator, reliably predicting shorter overall survival and poorer survival rates in colon cancer patients (Li et al., 2022a).

Functionally, USP30-AS1 exhibits tumor-suppressive properties by inhibiting colon cancer cell proliferation and metastasis through miRNA sponging mechanisms–a common regulatory strategy employed by lncRNAs in cancer biology (Pa et al., 2016). This lncRNA has been implicated in modulating the miR-299-3p/PTP4A1 axis in cervical cancer, while in colon cancer, it potentially interacts with miR-765. Elevated miR-765 levels, associated with tumor progression in non-small cell lung cancer and esophageal squamous cell carcinoma, inversely correlate with USP30-AS1 expression in colon cancer. Mechanistically, USP30-AS1 may suppress tumor growth by sequestering miR-765, thereby attenuating its oncogenic effects.

The tumor-promoting mechanisms of miR-765 involve multiple targets, including inositol polyphosphate 4-phosphatase type II (INPP4B) and interactions with LINC00511/Laminin Subunit Gamma 2 (LAMC2) in tongue squamous cell carcinoma (Xie et al., 2016; Ding et al., 2018). These findings suggest miR-765 may mediate USP30-AS1’s tumor-suppressive function in colon cancer through gene-specific regulatory pathways, though the precise molecular mechanisms require further elucidation.

#### 4.1.5 Acute myeloid leukemia (AML)

Acute myeloid leukemia (AML) is a malignant hematologic disorder originating from leukemia stem cells, characterized by complex pathogenic mechanisms. The long non-coding RNA USP30-AS1 has emerged as a critical oncogenic regulator in AML pathogenesis, exhibiting dual epigenetic and post-transcriptional regulatory functions. Hypomethylation at the Cg03124318 promoter site drives USP30-AS1 overexpression in AML, correlating with poor clinical outcomes through its anti-apoptotic and pro-survival effects on leukemic cells. Experimental evidence reveals that USP30-AS1 knockdown induces apoptosis while reducing cell viability, effects reversible through USP30 suppression, establishing a functional axis between these molecules.

Mechanistically, USP30-AS1 exerts cis-regulatory control over its neighboring USP30 gene, a key oncoprotein involved in mitochondrial homeostasis and lipid metabolism. This regulatory relationship modulates critical cellular processes including proliferation and apoptosis resistance. Furthermore, USP30-AS1 interacts with ANKRD13A to disrupt HLA-I protein trafficking from membrane to cytoplasm, potentially enabling immune evasion by reducing tumor antigen presentation. The lncRNA’s ability to influence chromatin remodeling adds another layer to its oncogenic profile, positioning it as a multifunctional regulator in leukemia progression.

These findings collectively identify USP30-AS1 as a master regulatory lncRNA in AML, orchestrating multiple oncogenic pathways through both gene-specific and broad epigenetic mechanisms. Its central role in maintaining malignant phenotypes and modulating tumor microenvironment interactions highlights its therapeutic potential as a multi-target intervention point for AML treatment strategies (Gu et al., 2018; Zhou et al., 2022).

#### 4.1.6 Cervical cancer (CC)

Cervical cancer (CC), one of the most common gynecological malignancies, ranks as the second most prevalent cancer and the fourth leading cause of cancer-related mortality in women worldwide (Chen M. et al., 2021). USP30-AS1 is overexpressed in CC tissues and cells, and its expression is associated with worse overall survival. Silencing USP30-AS1 significantly inhibits cell proliferation, migration, invasion, and tumor growth.

Both *in vitro* and *in vivo* studies demonstrate that USP30-AS1 enhances PTP4A1 expression by decoying miR-299-3p, thereby promoting the oncogenic potential of cervical cancer cells. The USP30-AS1/miR-299-3p/PTP4A1 axis may serve as both a molecular marker for cervical cancer progression and a potential therapeutic target (Chi et al., 2024).

Moreover, USP30-AS1 was found to modulate the expression of USP30 by sponging microRNA-2467-3p (miR-2467-3p) and recruiting the FUS RNA binding protein (FUS), thereby stabilizing β-catenin and activating the Wnt/β-catenin signaling pathway. These findings suggest that USP30-AS1 enhances CC cell growth and migration through the miR-2467-3p/FUS/USP30 axis, highlighting its potential as a biomarker for CC (Chi et al., 2024).

#### 4.1.7 Oral squamous cell carcinoma (OSCC)

Oral squamous cell carcinoma (OSCC) is a prevalent type of cancer affecting the head and neck, with smoking and alcohol being the primary risk factors (Bugshan and Farooq, 2020). Research indicates that USP30 is overexpressed in OSCC tissues and correlates with an unfavorable prognosis. Analysis of clinical data has shown that higher USP30 mRNA levels are present in OSCC tissues compared to normal adjacent tissues, with even greater expression in stage III patients than in those with stage I/II disease. Protein expression, as measured by western blot, also reveals increased USP30 levels in OSCC tissues, particularly in stage III patients (Zhang X. et al., 2022).

Studies have also shown that USP30 enhanced the deubiquitination of c-Myc, leading to an increase in c-Myc protein levels (Gomez et al., 2023). c-Myc works together with mutant β-catenin to promote hepatoblastoma (Zheng et al., 2017). In advanced OSCC, c-Myc collaborates with other oncogenes to advance cancer development (Pallavi et al., 2018). These discoveries highlighted a novel function of the USP30/c-Myc axis in OSCC, indicating that USP30 inhibited the ubiquitination and degradation of c-Myc, resulting in OSCC (Zhang X. et al., 2022).

Overall, USP30 upregulation was associated with poor prognosis and promoted OSCC progression (Zhang X. et al., 2022).

#### 4.1.8 Bladder urothelial carcinoma (BLCA)

Bladder urothelial carcinoma (BLCA) represents the most prevalent malignant neoplasm within the urinary system (Antoni et al., 2017), and the survival outcomes of BCLA patients are poor. USP30 is related to the occurrence and development of tumors, but current research may still be in the preliminary stage, and its specific role and mechanism in urothelial carcinoma of the bladder need further investigation.

Nevertheless, five autophagy related lncRNAs, including USP30-AS1, are potential prognostic and diagnostic biomarkers, as well as promising targets for BCLA therapy (Wan et al., 2021).

#### 4.1.9 Ovarian carcinoma (OC)

As one of the most fatal gynecological cancers, ovarian carcinoma poses a significant public health challenge globally (Ye et al., 2009; Ben-Arye et al., 2023; Geng et al., 2023). Currently, there is no direct link between USP30 and ovarian cancer. More experiments and clinical studies are needed to clarify the mechanism of USP30 in ovarian cancer and its potential as a therapeutic target. However, The USP30-AS1 gene is crucial for predicting the prognosis of ovarian cancer. Meanwhile, USP30-AS1 expression is positively correlated with several types of immune cells, such as Th1 cells, suggesting that this gene is crucial to shaping the immune landscape for ovarian cancer (Xiong et al., 2023).

#### 4.1.10 Melanomas

Melanomas are malignant neoplasms that can arise in various anatomical sites, including the skin, mucous membranes, uvea, and pia mater. Regarding USP30 and cutaneous melanoma, there is no direct evidence to suggest a direct connection between them. However, the risk models constructed using 15 autophagy-related long non-coding RNAs, including USP30-AS1, have important prognostic value and can provide autophagy-related therapeutic targets (Wan et al., 2021).

### 4.2 USP30 in neurodegenerative diseases

Neurodegenerative diseases, including Alzheimer’s, Parkinson’s, Huntington’s, and amyotrophic lateral sclerosis (ALS), are characterized by the gradual loss of cognitive and motor functions, affecting brain cells in a progressive manner (Dugger and Dickson, 2017).

In the context of neurodegenerative diseases, USP30 is considered a potential therapeutic target. Research has shown that inhibiting USP30 can enhance mitophagy, reducing oxidative stress and improving the health of neurons, especially in models of Parkinson’s disease. This suggests that USP30 inhibition could be a promising strategy for developing treatments aimed at preserving neuronal integrity and function in neurodegenerative conditions. Furthermore, studies have been the first to demonstrate the pathophysiological role of USP 30 in TBI (traumatic brain injury), providing new therapeutic strategies for the treatment of TBI.

#### 4.2.1 Parkinson disease (PD)

The Parkinson’s disease is a complex, age-related neurodegenerative disease caused by dopamine deficiency, as well as motor and nonmotor deficits (Simon et al., 2020). A hereditary early-onset form of the disease, known as autosomal recessive juvenile parkinsonism, represents up to 10% of all cases and has been associated with somatic mutations in genes that encode the PINK1 kinase and the E3 ligase parkin, among others (Kazi et al., 2025). In patients and models of Parkinson’s disease, mitophagy appears to be impaired, and this is associated with accelerated neurodegeneration (Liu et al., 2019). Inhibition of USP30 can enhance mitophagy, promoting the clearance of damaged mitochondria, and is being explored as a therapeutic strategy for conditions characterized by mitochondrial dysfunction.

Knockdown of USP30 in dopaminergic neurons confers protection against paraquat-induced toxicity *in vivo*, enhancing dopamine levels, motor function, and overall survival in flies (Zhang R. et al., 2022). Subsequently, Bingol et al. also found that USP30 deubiquitinase inhibits mitophagy by opposing parkin-mediated ubiquitination (Bingol and Sheng, 2016). The knockdown of USP15 and, to a lesser extent, USP30 of the *Drosophila* homologues of the deubiquitinases rescues mitophagy in parkin-deficient flies. These findings establish an essential role for parkin and PINK1 in regulating age-dependent mitophagy in *Drosophila in vivo* (Cornelissen et al., 2018).

Inhibition of USP30, either through an inhibitor or dominant-negative expression, elevated p-Ser65-ubiquitin levels and enhanced mitophagy in neuronal cell models, providing additional support for USP30 inhibition as a regulator of the mitophagy pathway (Tsefou et al., 2021). In dopaminergic neurons derived from Parkinson’s disease patients with Parkin RBR E3 ubiquitin protein ligase (PRKN) loss-of-function mutations, the benzosulphonamide compound 39 effectively restored mitophagy to near-normal levels by inhibiting USP30 (Rusilowicz-Jones et al., 2021). Under basal physiological conditions, F-box protein 7/nutcracker (FBXO7/ntc) acts to prime OMM proteins with ubiquitin counteracted by USP30 thereby establishing a critical surveillance checkpoint for mitochondrial protein import and damage control (Sanchez-Martinez et al., 2023).

In USP30 knockout mice, loss of USP30 leads to increased mitophagy, reduced phospho-S129 α-synuclein (αSyn), and attenuated SN dopaminergic neuronal loss induced by αSyn (Fang et al., 2023). Experiments have shown that an effective, selective, brain-penetrating USP30 inhibitor MTX115325 can significantly enhance mitochondrial autophagy, reduce phospho-S129 αSyn deposition, and restore dopamine levels (Okarmus et al., 2024).

Therefore, based on multiple studies, USP30 is a key regulatory target of Parkinson’s disease, and its mechanism of action can be summarized as the following points: Dysfunctional pairs of mitochondrial quality control in neurons are the central mechanism of PD pathogenesis, including idiopathic and genetic forms of PD (Borsche et al., 2021). USP30 is a key negative regulator of mitochondrial quality control, which can remove ubiquitin on some proteins outside the mitochondrial membrane, thereby inhibiting the PINK1-1/parkin-driven mitochondrial autophagy process (Pickles et al., 2018). In this process, PINK1 remains stable on mitochondria with decreased membrane potential to add a phosphate group to ubiquitin attached to proteins on the outer mitochondrial membrane. This subsequently results in the recruitment and activation of parkin, which collaborates with the autophagy machinery to mediate the lysosomal breakdown of damaged sections of the mitochondrial network (Narendra et al., 2009; Imberechts et al., 2022). In addition, USP30 may also be an amplifier of αSyn toxicity, and USP30 deletion or inhibition can significantly reduce phosphorylation-S129 αSyn deposition, restore dopamine levels, and prevent neuronal loss.

In summary, inhibiting USP30, enhancing mitophagy, clearing damaged mitochondria, and protecting dopaminergic neurons constitute a promising disease-modifying strategy for Parkinson’s disease.

#### 4.2.2 Alzheimer disease (AD)

*In vivo* experiments demonstrated that miR-137-5p enhances the cognition and mobility of AD mice, significantly reducing Aβ1-42 deposition, Tau hyperphosphorylation, and neuronal apoptosis within the hippocampus and cortex regions. Mechanistically, miR-137-5p significantly suppresses USP30 levels in mice, though USP30 overexpression partially buffers against miR-137-5p-induced AD symptom improvement. MiR-137-5p, through its regulation of USP30 downregulation, represents a novel and promising therapeutic target for AD. USP30’s function and regulatory mechanisms in AD progression, however, remain unclear (Jiang et al., 2023).

#### 4.2.3 Traumatic brain injury (TBI)

USP30 protein expression is upregulated in neurons under TBI conditions in both human patients and mouse models. Neuron-specific deletion of USP30 inhibits neuronal apoptosis, decreases lesion volume, and alleviates neurological deficits following TBI; Ablation of USP30 reduces oxidative stress and mitochondrial quality control caused by TBI. This is the first study to demonstrate that USP30 plays a pathophysiological role in TBI, revealing a novel therapeutic approach (Wu et al., 2023).

### 4.3 USP30 in pulmonary diseases

In addition to neurodegenerative diseases, mitophagy also affects pulmonary diseases. In aging-associated pulmonary disorders, mitochondrial damage accumulates. Mitochondrial dysfunction, resulting from the accumulation of damage, is a key factor in the development of pulmonary disorders associated with aging. Within the multitude of pathways that preserve mitochondrial health, dysregulated mitophagy, specifically involving the PINK1-PARK2 pathway, plays a significant role in determining cellular outcomes such as apoptosis, senescence, and the differentiation of myofibroblasts. This is particularly relevant in the pathogenesis of chronic obstructive pulmonary disease (COPD) and idiopathic pulmonary fibrosis (IPF). As part of the pathogenesis of chronic obstructive pulmonary disease, other studies have shown reduced PARK2 expression levels in association with insufficient mitophagy and epithelial cell senescence (Siekacz et al., 2021). Disorganized fibrosis development in IPF might be caused by reduced PARK2-mediated insufficient mitophagy (Ito et al., 2015).

The inhibition of USP30 can promote PINK1/Parkin-mediated mitophagy, which may be beneficial for treating pulmonary diseases related to the aforementioned mitochondrial dysfunction. And USP30 inhibitors are already in preclinical stages in treating lung diseases, suggesting they may have potential in treating lung diseases. For example, pirfenidone, a drug that has been approved for the treatment of IPF (Noble and Homer, 2004).

### 4.4 USP30 in cardiovascular diseases

Although the research on USP30 in cardiovascular diseases is relatively limited compared to neurodegenerative diseases and cancer, emerging studies suggest its potential involvement. In myocardial cells, USP30 has been shown to negatively regulate mitophagy and accelerate myocardial cell senescence through antagonism of Parkin (Pan et al., 2021). This indicates that USP30 may play a role in age-related cardiovascular pathologies.

As mitochondria play a crucial role in cardiac function, and USP30 is involved in mitochondrial quality control and autophagy regulation, it is possible that dysregulation of USP30 could contribute to cardiovascular diseases. However, more research is needed to fully understand the specific mechanisms and significance of USP30 in cardiovascular diseases.

## 5 Advances in targeting USP 30 inhibitors

**FIGURE 4:**
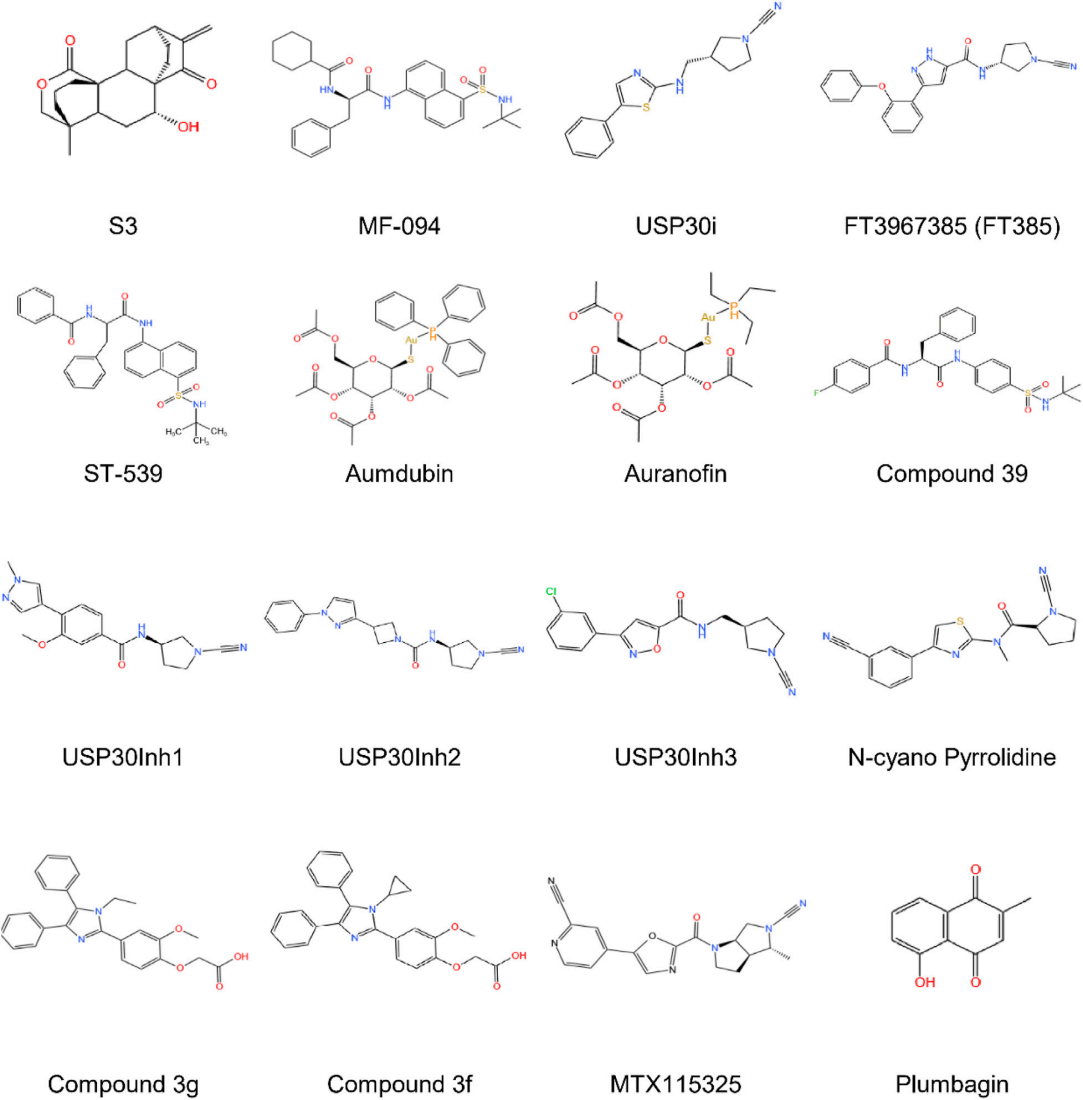
The development process and chemical structure of USP30 inhibitors.

Recent advancements in the development of USP30 inhibitors have demonstrated promising therapeutic potential through modulation of mitochondrial dynamics and protein ubiquitination pathways. Researchers have identified multiple inhibitors targeting this deubiquitinating enzyme, including diterpenoid-derived 15-oxospiramilactone (S3), MF-094, USP30i, FT3967385, ST-539, aumdubin, compound 39, USP30Inh1-3 *etc.*, each exhibiting distinct mechanisms of action and biological effects (Figure 4; Table 2).

**TABLE 2:** The development process and characterization of USP30 inhibitors.

Inhibitor	Development process	IC50	Cellular effect	References
S3	15-oxyspironolactone derived from the diterpenoids	None reported	Increase the ubiquitination of Mfn1 and Mfn2 without affecting their protein levels	Yue et al. (2014)
MF-094	A hit-to-lead optimization	0.12 μM	Accelerate mitophagy	Kluge et al. (2018), Li et al. (2022b)
USP30i	Unknown	2.45 μM	Increase the ubiquitination of TOM20	Phu et al. (2020)
FT3967385 (FT385)	A modified N-cyano pyrrolidine tool compound highly-selective USP30 inhibitor	1 nM	Recapitulate the promoting effects of USP30 depletion on mitophagy and show similar elevation of the ubiquitinated TOM20	Rusilowicz-Jones et al. (2020)
ST-539	A racemic phenylalanine derivative reported in previous studies	None reported	Modulate PINK1/Parkin-dependent mitophagy and efficiently induces cardiac mitophagy	Luo et al. (2021)
Aumdubin	Synthesized by the replacement of TPP group with ethyl group from a derivative of auranofin	None reported	Induce apoptosis by increasing the ubiquitination and mitochondrial location of Bax protein	Yan et al. (2021)
Compound 39	Designed to feature the previously reported compounds	20 nM	Increase mitophagy and basal pexophagy; highly selective for neuronal USP30	Rusilowicz-Jones et al. (2021)
Compound 3g	From an imidazole series of ligands	5.12 μM	inhibit apoptosis on SH-SY5Y neuroblastoma cells against dynorphin A (10 μM) treatment	Mandal et al. (2022)
Compound 3f		8.43 μM		
USP30Inh-1	Based on compound structures published in previous patents	15–30 nM	Potently inhibit USP30-mediated cleavage of Ub-Rho110; increase mitophagy in the SH-SY5Y neuronal cells	Tsefou et al. (2021)
USP30Inh-2				
USP30Inh-3				
Q14	A peptide inhibitor derived from the TM domain of USP30; a novel autoinhibitory mode	57.2 nM ±4.77	Degrade OMM proteins, promote mitochondrial fission, and trigger mitophagy	Wang et al. (2024)
MTX115325	Developed by Mission Therapeutics	12 nM	Prevent dopaminergic neuronal loss and dopamine depletion in an α-synuclein-based PD mouse model	Fang et al. (2023)
Chaihu Shugan Powder	Originated from Jingyue Quanshu	None reported	Effectively suppress the process of PINK1/Parkin- mediated mitophagy	Yao et al. (2023)

The diterpenoid derivative S3 (15-oxospiramilactone, 330 Da) binds specifically to cysteine 77 in USP30’s catalytic domain, effectively inhibiting its deubiquitinase activity (Zhuang et al., 2022). This interaction increases non-degradative ubiquitination of mitochondrial fusion proteins MFN1 and MFN2 without altering their expression levels. Notably, S3 demonstrates concentration-dependent effects: while higher concentrations induce apoptosis *via* Wnt pathway inhibition (Wang et al., 2011) and Bim upregulation, lower concentrations (e.g., 2 μM) selectively promote mitochondrial fusion through USP30 inhibition. This dual functionality positions S3 as a potential therapeutic candidate for disorders involving mitochondrial dynamics, particularly in retinal ganglion cells where USP30 knockdown enhances MFN1/2-mediated fusion processes (Yue et al., 2014).

MF-094, a highly selective USP30 inhibitor (IC_50_ = 0.12 μM), demonstrates mitophagy-enhancing effects through accelerated clearance of 5-bromo-2-deoxyuridine (BrdU) from mitochondrial DNA. Preclinical studies in diabetic rat models reveal that MF-094 treatment reduces NLRP3 inflammasome activity (*via* caspase-1 p20 downregulation) and improves wound healing, suggesting therapeutic utility for diabetic complications such as foot ulcers (Kluge et al., 2018; Li et al., 2022b).

USP30 inhibitors (USP30i) enhance TOM20 ubiquitination, a hallmark of mitophagy activation. Phu et al. employed mass spectrometry to profile ubiquitinated substrates in USP30-inhibited or knockout HEK293 cells, identifying off-target interactions with DUBs such as desumoylating isopeptidase 2 (DESI2), ataxin 3 (ATXN3), ubiquitin-specific proteases 4 (UBP4), UBP45, and UBP47. These findings suggest USP30 may counterbalance ubiquitin ligase activity in localized protein quality control, analogous to USP14 and ubiquitin C-Terminal hydrolase L5 (UCHL5) mechanisms (Phu et al., 2020).

Rusilowicz-Jones et al. introduce FT3967385 (hereafter FT385), a modified N-cyano pyrrolidine tool compound highly-selective USP30 inhibitor. To evaluate the inhibitor’s selectivity among the USP enzyme family, Rusilowicz-Jones and colleagues employed the Ubiquigent DUB profiler screen to evaluate inhibitory effects on a wide range of USP enzymes. The inhibitor demonstrated high selectivity for USP30 at concentrations up to 200 nM USP6, which is associated with the plasma membrane, was the only other member of the family to show significant inhibition. It was demonstrated that FT385 can recapitulate the effects of depleting USP30 on mitophagy and increase levels of the ubiquitinated TOM20 in the same way as USP30 depletion. Proteomics analysis of SHSY5Y neuroblastoma cells with USP30 genetic knockout or FT385 treatment revealed off-target effects (Rusilowicz-Jones et al., 2020).

The mitochondrial-targeted compound ST-539 exhibits favorable pharmacokinetic properties with minimal cellular toxicity. Its pharmacological inhibition of USP30 modulates PINK1/Parkin-dependent mitophagy pathways, showing particular promise for cardiac applications. Current research focuses on optimizing dosing regimens to balance tissue-specific effects and therapeutic outcomes, though precise parameters for clinical translation require further investigation (Luo et al., 2021).

Aumdubin, a gold(I)-based auranofin derivative modified with an ethyl group substitution, demonstrates enhanced DUB inhibitory activity compared to its parent compound. Although showing affinity for multiple DUBs including USP14, USP10, and UCHL5, functional studies reveal its cytotoxic effects in lung cancer cells specifically depend on USP30 inhibition. This selective activity induces BAX-dependent apoptosis, suggesting therapeutic potential for malignancies while highlighting the ongoing challenge of achieving absolute DUB specificity in inhibitor design (Yan et al., 2021).

Compound 39, a benzosulphonamide derivative, demonstrates high potency and selectivity for neuronal USP30 through slow, tight-binding inhibition (O'Brien et al., 2023). In cellular models of Parkinson’s disease with PRKN mutations, this compound restores mitophagy to near-normal levels while enhancing ubiquitination markers (TOM20, SYNJ2BP) and phosphoubiquitin accumulation. Additional effects on pexophagy in U2-OS cells suggest broader applications in organelle quality control mechanisms (Rusilowicz-Jones et al., 2021).

The USP30Inh series (1–3), designed from patented scaffolds (WO 2016/156816; WO 2017/103614), demonstrated potent USP30 inhibition (IC50 15–30 nM) using Ub-Rho110 cleavage assays. While exhibiting >40 DUB selectivity at 1μM, higher concentrations (10 μM) revealed off-target effects against USP6, USP21, and USP45. All three compounds enhanced mitophagy in neuronal SHSY5Y cells, though their shared cyanoamide group necessitates rigorous assessment of mitochondrial toxicity in biological applications (Tsefou et al., 2021).

Subhankar Mandal et al. identified imidazole-based USP30 inhibitors (3g: IC50 = 5.12μM; 3f: IC50 = 8.43 μM) synthesized *via* a Zn (l-proline)_2_-catalyzed method. Compound 3g demonstrated neuroprotective efficacy by attenuating dynorphin A-induced apoptosis in neuroblastoma cells, highlighting its potential for treating neurodegenerative disorders and kidney injury (Mandal et al., 2022).

Qin et al. identified a novel peptide (Q14) derived from the transmembrane (TM) domain of USP30, which directly targets mitochondrial-anchored USP30. Using a Cys-Met bis-alkylated sulfonium tethered peptide, they further investigated the Q14 peptide-USP30 binding site through proximity-promoted protein labeling. The Q14 peptide readily crosses cell membranes, binds with mitochondrial-anchored USP30 directly, degrades OMM proteins, activates mitophagy, and promotes mitochondrial fission. Moreover, the Q14 peptide can bind to LC3 and connect mitochondrial-bound USP30 to the phagophore membrane, speeding up mitophagy. The Q14 peptide weakly inhibits USP2, USP7, and USP8, indicating it may regulate USP30 activity through distinct allosteric mechanisms rather than mere steric hindrance. Using a novel autoinhibitory (a mechanism proteins use to guard against spurious activation) mode, allosteric regulation at the tip of the fingers subdomain, Q14 peptide inhibits the USP30 activity in an autoinhibitory mode. Investigating allosteric mechanisms that regulate autoinhibition might be beneficial in identifying effective inhibitors for USP30 or other DUBs (Bingol et al., 2014).

MTX115325, a CNS-penetrant USP30 inhibitor with oral bioavailability, prevented dopaminergic neuron loss in α-synuclein Parkinson’s disease models, underscoring its therapeutic promise (Fang et al., 2023).

Further supporting USP30’s role in mitophagy regulation, Wang et al. (2023) observed inverse expression patterns between Parkin and USP30 *in vivo*. This relationship is exploited by Chinese medicine compound Chaihu Shugan Powder (CHSGP), a traditional formulation shown to suppress PINK1/Parkin-mediated mitophagy in interstitial Cajal cells *via* USP30 inhibition. These findings propose CHSGP as a potential intervention for functional dyspepsia by modulating mitochondrial quality control pathways (Wang et al., 2024).

Plumbagin, a natural product DUB inhibitor, uniquely induces glutathione peroxidase 4 (GPX4) ubiquitination and degradation in hepatocellular carcinoma cells *via* selective USP31 inhibition. Notably, USP30 and other DUBs (CYLD, USP1, UCHL family) showed no involvement in this pathway, emphasizing plumbagin’s distinct mechanism (Yao et al., 2023).

Current challenges in USP30 inhibitor development include achieving sufficient target specificity, optimizing pharmacokinetic profiles for *in vivo* applications, and understanding tissue-specific responses to enzymatic inhibition. The diversity of molecular scaffolds under investigation—ranging from natural product derivatives to synthetic peptides—reflects growing recognition of USP30’s central role in mitochondrial homeostasis and disease pathogenesis. Continued progress in structural characterization and mechanism elucidation will be critical for translating these findings into clinically viable therapies. Future research also should foucus on investigating the potential off-target effects of such inhibitors, especially in clinical settings. Moreover, expanding investigations into USP30’s role in understudied pathologies—particularly neuro-muscular disorders and primary mitochondrial diseases where mitophagy defects are implicated—may reveal new druggable pathways.

## 6 Conclusion

**FIGURE 5:**
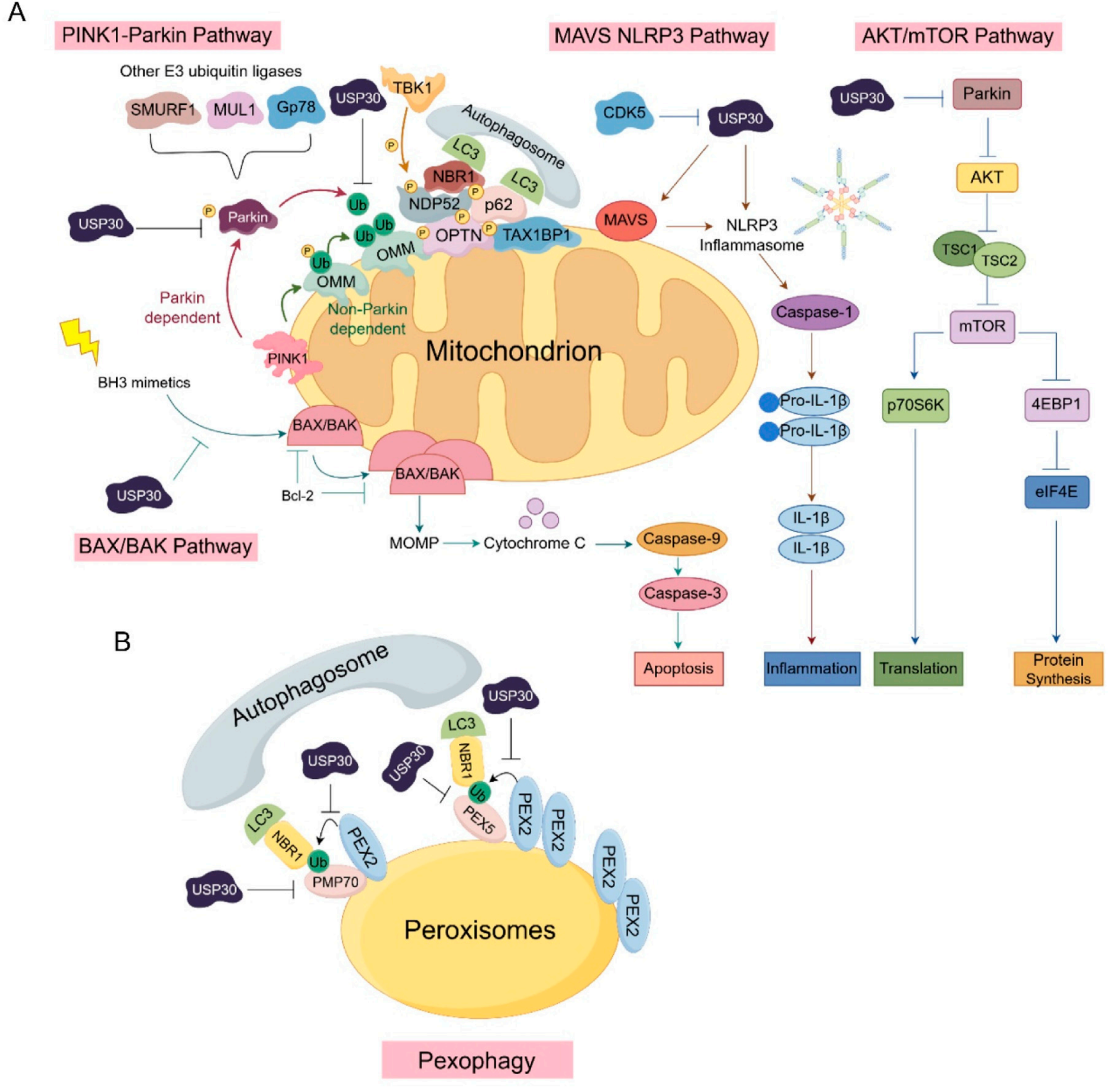
The involvement of USP30 in various signaling pathways: **(A)** The critical components and cascade reactions of signal transduction within the PINK1-Parkin, MAVS-NLRP3, AKT/mTOR, and BAX/BAK signaling pathways in mitochondria. **(B)** The role of USP30 in signaling pathways associated with peroxisomes.

USP30, a 517-amino acid protein with six potential isoforms, distinguishes itself from other ubiquitin-specific proteases through its unique catalytic triad (Cys77, His452, and Ser477) and molecular architecture that confers preferential cleavage specificity for K6-linked ubiquitin chains. This deubiquitinating enzyme plays multifaceted roles in critical cellular processes, including the regulation of mitophagy and mitochondrial dynamics, pexophagy, apoptosis, and cell death regulation. Emerging evidence underscores its significant involvement in various pathological conditions, particularly demonstrating abnormal expression patterns across multiple cancer types that correlate with tumor progression and clinical outcomes. Notably, USP30’s neuroprotective potential has been demonstrated through both genetic knockout and pharmacological inhibition studies, which revealed its capacity to enhance mitophagy and mitigate α-synuclein toxicity (Figures 5A,B).

The enzyme’s disease-associated roles extend beyond oncology to neurodegenerative disorders, pulmonary diseases, and inflammatory conditions, positioning USP30 as a compelling therapeutic target. Future investigations should prioritize elucidating the mechanistic details of USP30’s involvement in disease pathogenesis while exploring therapeutic strategies through activity modulation. One of the main challenges in targeting USP30 for treatment is understanding its complex role in different cellular processes. USP30 is involved in multiple pathways, such as mitochondrial quality control, autophagy, and apoptosis, and its dysregulation can have diverse effects depending on the cell type and disease context. Another challenge is the development of specific and effective inhibitors. This could involve the use of advanced techniques such as structure-based drug design, high-throughput screening, and *in-vivo* models to optimize inhibitor properties. Although some inhibitors have been identified, there is still a need to improve their selectivity, potency, and pharmacokinetic properties. For example, the high flexibility of USP30’s catalytic site and its dependence on covalent interaction with the catalytic cysteine present challenges in discovering suitable small-molecule inhibitors (Anbazhagan et al., 2025). Additionally, the potential off-target effects of inhibitors need to be carefully evaluated to ensure their safety and efficacy in clinical applications.

Potential breakthroughs in USP30-targeted therapies may come from the development of more specific and effective inhibitors. For example, the use of nano delivery systems to deliver USP30 inhibitors, as demonstrated in oral squamous cell carcinoma, could improve the therapeutic efficacy by enhancing the delivery and targeting of inhibitors to cancer cells (Zhang X. et al., 2022). In neurodegenerative diseases, the development of brain-penetrant USP30 inhibitors with good drug-like properties may slow down or reverse the progression of diseases like Parkinson’s disease by enhancing mitophagy and improving mitochondrial function, leading to significant breakthroughs.

Future research on USP30 should prioritize the development of highly specific inhibitors, leveraging structural biology and targeted screening to overcome the challenge of selectivity within the conserved USP deubiquitinase family. Damianou et al. have developed a novel technology, offering provides a strong framework for identifying DUB-substrate interactions and improving our comprehension of ubiquitin-regulated pathways. The proximal-ubiquitomics approach could detect changes in ubiquitination events occurring in the proximity of USP30, facilitating the recognition of specific ubiquitination occurrences that could be used as strong biomarkers of USP30 inhibition. More broadly, proximal-ubiquitomics could be applied more widely, not only for DUBs but also for discovering E3 substrates. This approach could assist in designing proteolysis-targeting chimeras (PROTACs), molecular glues, and deubiquitinase-targeting chimeras (DUBTACs) with potential for translation (Damianou et al., 2025).

Moreover, combination therapy approaches warrant systematic exploration, particularly investigating potential synergies between USP30-targeted agents and established treatment modalities such as immunotherapy or chemotherapy. Such combinatorial strategies could enhance therapeutic effectiveness while potentially mitigating drug resistance mechanisms. These research avenues collectively hold significant promise for translating our growing understanding of USP30 biology into novel therapeutic interventions across multiple disease domains.

## Glossary

USP30: ubiquitin specific protease 30
Ub: ubiquitin
DNA: deoxyribonucleic acid
DUBs: deubiquitylating enzymes
USPs: ubiquitin-specific proteases
UCHs: ubiquitin carboxy-terminal hydrolases
OTUs: ovarian-tumor proteases
JAMMs: JAMM/MPN domain-associated metallopeptidases
MCPIP: ubiquitin carboxy-terminal hydrolases
DRP1: dynamin-related protein 1
AKT/mTOR: protein kinase B/mechanistic target of rapamycin
TOMM20: translocase of outer mitochondrial membrane 20
PINK1: PTEN-induced putative kinase 1
HSP90: Heat Shock Protein 90
OTUB1: OTU domain-containing ubiquitin aldehyde-binding protein
MFN: mitofusin
OMM: outer mitochondrial membrane
siRNA: small interfering RNA
PROTACs: proteolysis-targeting chimeras
DUBTACs: deubiquitinase-targeting chimeras
GTPase: GTP hydrolase
GNPAT: Glyceronephosphate O-acyltransferase
HCC: hepatocellular carcinoma
OGDR: oxygen-glucose deprivation/reperfusion
RGCs: retinal ganglion cells
ROS: reactive oxygen species
PEX2: peroxisomal biogenesis factor 2
PMP70: 70 kDa Peroxisomal Membrane Protein
PBDs: peroxisome biogenesis disorders
MOMP: mitochondrial outer membrane permeabilization
BAX: BCL2 associated X
BAK: BCL2 antagonist/killer 1
4EBP1: Eukaryotic translation initiation factor 4E-binding protein 1
P70S6K: Ribosomal protein S6 kinase
Sevo: Sevoflurane
NLRP1: NOD-like-receptor containing a pyrin domain 1
PD: Parkinson’s disease
MPTP/MPP+: 1-methyl-4-phenyl-1,2,3,6-tetrahydropyridine/1-methyl-4-phenylpyridinium
CDK5: cyclin-dependent kinase 5
MAVS: mitochondrial antiviral signaling protein
BC: breast cancer
GBM: glioblastoma
AML: acute myeloid leukemia
CC: cervical cancer
OSCC: oral squamous cell carcinoma
BLCA: bladder urothelial carcinoma
TOMM40: translocase of outer mitochondrial membrane 40
